# High-Intensity Statin With Severe Consequences: A Case of Non-autoimmune Rosuvastatin-Induced Myonecrosis

**DOI:** 10.7759/cureus.30080

**Published:** 2022-10-08

**Authors:** Mariam Hassan, Bryan Nguyen, Kristen Helmsdoerfer, Sahithi Nadella, Aymara Chang Lopez

**Affiliations:** 1 Internal Medicine, Texas Tech University Health Sciences Center El Paso, El Paso, USA; 2 Internal Medicine, Paul L. Foster School of Medicine, El Paso, USA

**Keywords:** atherosclerotic cardiovascular disease, cardiovascular prevention, elevated creatine kinase, aseptic myonecrosis, non-traumatic rhabdomyolysis, moderate-statin therapy, statin-induced myopathy, myalgia

## Abstract

Statins constitute a cornerstone in the primary and secondary prevention of atherosclerotic cardiovascular disease (ASCVD). The routine use of these lipid-lowering agents may lead to unintentional neglect of their well-known myotoxic properties. We report the case of a 77-year-old female with a two-year history of rosuvastatin use who presented with progressive bilateral upper and lower extremity muscular weakness for one week, which improved upon discontinuation of her long-term statin therapy. The authors aim to draw attention to this potentially underdiagnosed cause of disability. It is imperative that clinicians are able to appreciate the myopathic spectrum of statin therapy, irrespective of the duration of use. Myonecrosis, in particular, can progress to rhabdomyolysis, leading to irreversible renal dysfunction, electrolyte abnormalities, and subsequent cardiac dysrhythmias. Ultimately, statin-induced myopathy may significantly hinder activities of daily living and impair quality of life. It is, however, a reversible condition if diagnosed and appropriately managed early on. Clinicians are encouraged to acquaint themselves with the symptomatology and relevant laboratory values that commonly accompany this acute condition.

## Introduction

Statins or 3-hydroxy-3-methylglutaryl coenzyme A (HMG-CoA) reductase inhibitors are potent low-density lipoprotein (LDL)-lowering drugs that are widely used in clinical practice. A multitude of clinical trials has demonstrated the significant benefits among men and women, middle-aged and older persons, in the setting of primary and secondary prevention of atherosclerotic cardiovascular disease (ASCVD) [1]. A reduction in peripheral vascular disease, coronary heart disease, acute coronary syndrome, revascularization procedures, and stroke has been documented [1]. In 2019, the American College of Cardiology (ACC) /American Heart Association (AHA) guidelines on the ‘primary prevention of cardiovascular disease’ encouraged that both moderate and high-intensity statin therapy be employed given the direct correlation between reduced LDL cholesterol (LDL-C) levels and improved ASCVD outcomes [2]. As of August 2022, the United States Preventive Services Task Force (USPSTF) recommends that adults without a history of cardiovascular disease (CVD) use a low to moderate-intensity statin in the prevention of CVD events and mortality when the following criteria have been met: (a) aged 40-75 years, (b) have one or more CVD-related risk factors, and (c) have a calculated 10-year risk of cardiovascular event of 10% or greater (Grade B recommendation) [3].

Statins are one of the most prescribed medications in the United States. The initiation of statin therapy, however, should be a shared decision between the physician and the patient. The authors encourage clinicians to have a high index of suspicion for potential statin-induced myopathy in patients presenting with muscle pain or weakness, considering the prevalent use of these medications. However, it is imperative that other rheumatologic and neurologic causes of pain and weakness be ruled out prior to discontinuation of statin therapy given their demonstrated benefits. Differential diagnoses include, but are not limited to, muscle strain, tendonitis, radiculopathy, or osteoarthritis. Elevation of muscle enzymes may prompt the immediate cessation of lipid-lowering therapy, once its benefits are weighed against the risk of furthering myotoxicity.

Here, we report the case of a 77-year-old female who presented with bilateral upper and lower extremity muscular weakness for one week and an elevated creatine kinase (CK) level, all of which gradually improved with the discontinuation of her long-term statin therapy.

## Case presentation

A 77-year-old Hispanic female presented with progressive bilateral upper and lower extremity muscular weakness for one week. The patient stated that, for the last week, she had low energy levels with significant discomfort in her lower extremities that prevented her from performing basic activities of daily living. The patient explained that she first noticed weakness in her legs when attempting to get in a car. She was still able to comb her hair and use utensils, but with some difficulty. She denied having double vision, dysarthria, dysphagia, or sensory loss.

Past medical history was notable for diabetes mellitus, hypertension, coronary artery disease, heart failure with preserved ejection fraction, and rate-controlled atrial fibrillation. Home medications included insulin glargine, metformin, amlodipine, metoprolol, furosemide, apixaban, amiodarone 100 mg twice daily, and rosuvastatin 40 mg every night for the last two years.

On admission, the patient's vital signs were within normal limits. She had a normal range of motion in all joints. There was no erythema, swelling, tenderness to palpation of her extremities, or muscle atrophy. The speech was clear and coherent. Ocular movements were full and without nystagmus. The face was symmetric and without weakness. The tongue was midline. Motor exam showed proximal weakness in both upper (4/5) and lower (3/5) limbs, especially of the deltoids, biceps, triceps, iliopsoas, and adductors. She was unable to rise from a chair with her arms crossed. Sensation remained normal. Reflexes were 1+.

**Figure 1:**
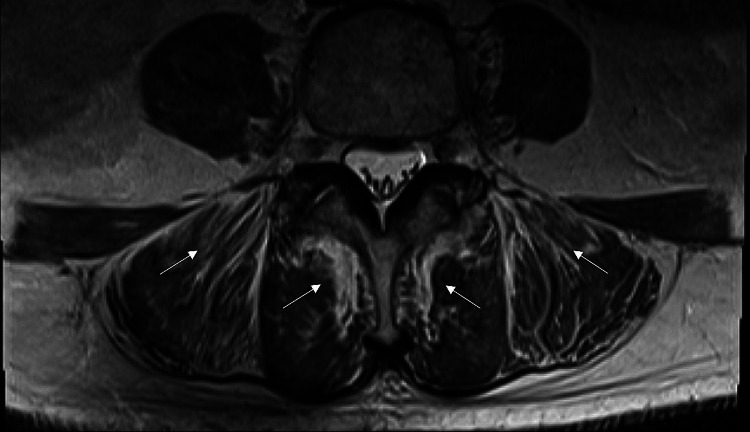
Inflammatory Myopathy/Myositis MRI Spine Lumbar with contrast exhibiting diffuse inflammatory myopathy/myositis of the bilateral iliacus, gluteus, and lower posterior paraspinal muscles bilaterally (arrows).

An MRI of the lumbar spine (Figure 1) with contrast showed diffuse inflammatory myopathy/myositis involving the bilateral visualized iliacus, gluteus, and lower posterior paraspinal muscles bilaterally, with degenerative changes of the lumbar spine most notable at L3-4 and L4-5. The differential diagnoses included toxic or autoimmune inflammatory myositis, nerve compression, and malignancy.

Laboratory work-up unveiled: erythrocyte sedimentation rate (ESR) 46, aspartate aminotransferase (AST) 373, alanine aminotransferase (ALT) 222, lactate dehydrogenase (LDH) 604, CK 9,839, and aldolase 46.9. Serum cobalamin, folate, and thyroid stimulating hormone (TSH) levels were within normal levels. Kidney function remained normal and a urinalysis was negative for myoglobinuria. Rapid plasma reagin (RPR) was non-reactive, antinuclear antibody (ANA), anti-double stranded DNA (anti-dsDNA), and the extended myositis panel, which included HMG-CoA reductase, were also negative.

The patient’s daily dose of rosuvastatin was quickly discontinued. Her muscle strength gradually improved and she began to ambulate with minimal assistance.

## Discussion

Although statins are generally well-tolerated, the most commonly reported adverse effect is muscular-related pain [4]. A spectrum of myopathies is observed with statin use, ranging from mild myalgic symptoms to rhabdomyolysis. For standardization purposes, the definitions in Table 1 are adopted.

**Table 1:** Spectrum of statin-associated muscle adverse events Based on the spectrum of statin-associated muscle adverse events delineated by the National Lipid Association's Muscle Safety Expert Panel (2014) [4] CK: creatine kinase

Muscle adverse events
Myalgia: Muscle pain or tenderness
Myopathy: Muscle weakness unrelated to pain, with or without an elevated creatine kinase (CK) level
Myositis: Muscle inflammation
Myonecrosis: Elevation in muscle enzymes
Rhabdomyolysis: Myonecrosis (specifically, a CK level greater than 10 times the upper limit of normal) with myoglobinuria or acute renal failure

The Effect of Statins on Muscle Performance (STOMP) double-blind clinical trial investigated the incidence of statin-associated muscle complaints in statin-naive subjects [4]. The study found that 9.4% of patients taking atorvastatin 80 mg/day over six months developed myalgia compared to 4.6% of placebo patients [4]. A retrospective study conducted in 2005 concerning statin-induced myopathy highlighted that the mean duration of statin therapy prior to symptom onset was 6.3 (9.8) months [5]. The patients experienced full resolution upon cessation of statin therapy, although 13% of the patients required hospitalization for rhabdomyolysis [5].

Myonecrosis is a rare complication of statin therapy leading up to rhabdomyolysis, with an estimated prevalence of 0.1% [6]. It is defined by the Statin Muscle Safety Task Force as an elevation of serum CK at at least three times greater than baseline [4]. Our patient met the criteria for moderate-to-severe statin-induced myonecrosis following a two-year history of rosuvastatin use.

High-intensity statins (e.g. atorvastatin and rosuvastatin) are more likely to induce myopathic symptoms compared to moderate-intensity statins [7]. High-intensity statins are lipophilic and predominantly metabolized by the CYP3A4 system [7]. Concomitant CYP inhibitor use increases the serum concentration of CYP substrates, potentiating their toxic effects. A thorough literature review revealed a paucity of studies concerning non-autoimmune moderate-intensity statin-induced myonecrosis.

The patient’s concomitant use of amiodarone, a well-known CYP450 inhibitor, and amlodipine, a competing CYP3A4 substrate, convoluted this case presentation. Amiodarone has also been reported to induce neuromyopathy characterized by normal serum CK values [8]. This patient’s markedly elevated CK insinuated that the culprit of her pathology was statin use. We postulate that the combination of minor metabolism inhibition by amiodarone and competing amlodipine, in conjunction with her maximally dosed rosuvastatin, led to a toxic accumulation of the statin; segueing across the spectrum to myonecrosis. A final point to highlight is that the patient tested negative for HMG-CoA reductase antibodies, ruling out an autoimmune subtype of statin-induced myonecrosis and further implicating direct toxicity.

By reflecting on our limitations in addressing this particular case scenario, we encourage readers to have a high index of suspicion for statin-induced myonecrosis when patients complain of muscle pain or weakness, irrespective of statin intensity or the duration of use. This is especially true in patients with conditions that warrant statin therapy, such as diabetes mellitus, hyperlipidemia, and CVD. While the symptoms of statin-induced myopathies can be reversible upon statin discontinuation, they are incredibly distressing for patients. The symptoms may take weeks to resolve, necessitating prompt recognition of this condition, and removal of the offending agent.

## Conclusions

Statins are notorious for evoking myopathies that vary in severity. The case reported here draws attention to myonecrosis and its high risk for progression to rhabdomyolysis. Despite this potential severe adverse effect, statin use prevails given documented benefits in primary and secondary prevention of ASCVD. Management encompasses discontinuation of statin therapy, a thorough medicine reconciliation, and obtaining an HMG-CoA reductase IgG level to rule out an underlying autoimmune phenomenon that is essential in guiding management.

Ultimately, a delayed diagnosis of statin-induced myonecrosis may lead to advanced disease, hospitalization, treatment of rhabdomyolysis-related complications, and slow recovery to the patient's baseline. Patients should be routinely reminded to report muscle-related discomfort, weakness, and/or urine discoloration regardless of statin intensity or duration of treatment. Scheduled dose-response and tolerance assessments following six to eight weeks of therapy are advised by the ACC/AHA.
